# NFIL3 contributes to cytotoxic T lymphocyte-mediated killing

**DOI:** 10.1098/rsob.230456

**Published:** 2024-02-28

**Authors:** Tiphaine Douanne, Katharina Strege, Martin Del Castillo Velasco-Herrera, Adam M. Rochussen, David J. Adams, Gillian M. Griffiths

**Affiliations:** ^1^ Cambridge Institute for Medical Research, Keith Peters Building, Cambridge CB2 0XY, UK; ^2^ Experimental Cancer Genetics, The Wellcome Trust Sanger Institute, Hinxton, Cambridge, UK

**Keywords:** NFIL3, cytotoxic T cell, T-cell-mediated killing, perforin, granzyme, autoimmunity

## Abstract

Cytotoxic T lymphocytes (CTLs) are key effectors of the adaptive immune system that recognize and eliminate virally infected and cancerous cells. In naive CD8^+^ T cells, T-cell receptor (TCR) engagement drives a number of transcriptional, translational and proliferation changes over the course of hours and days leading to differentiation into CTLs. To gain a better insight into this mechanism, we compared the transcriptional profiles of naive CD8^+^ T cells to those of activated CTLs. To find new regulators of CTL function, we performed a selective clustered regularly interspaced short palindromic repeats (CRISPR) screen on upregulated genes and identified nuclear factor IL-3 (NFIL3) as a potential regulator of cytotoxicity. Although NFIL3 has established roles in several immune cells including natural killer, Treg, dendritic and CD4^+^ T cells, its function in CD8^+^ CTLs is less well understood. Using CRISPR/Cas9 editing, we found that removing NFIL3 in CTLs resulted in a marked decrease in cytotoxicity. We found that in CTLs lacking NFIL3 TCR-induced extracellular signal-regulated kinase phosphorylation, immune synapse formation and granule release were all intact while cytotoxicity was functionally impaired *in vitro*. Strikingly, NFIL3 controls the production of cytolytic proteins as well as effector cytokines. Thus, NFIL3 plays a cell intrinsic role in modulating cytolytic mechanisms in CTLs.

## Graphical abstract

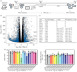

## 1. Introduction

CD8^+^ cytotoxic T lymphocytes (CTLs) are key effectors of the adaptive immune response that precisely recognize and eliminate virally infected and cancerous cells. In naive CD8^+^ T cells, T-cell receptor (TCR) engagement induces a number of transcriptional, translational and proliferation changes over the course of hours and days leading to differentiation into CTLs [1,2]. TCR ligation of differentiated CTLs drives a rapid response and the formation of a transient area of plasma membrane specialized in signalling and polarized secretion, termed the immune synapse [3]. CTLs undergo rapid rearrangements in microtubule and actin cytoskeletons as the centrosome and microtubule network polarize towards the synapse and cortical actin is transiently depleted [4–7]. This tightly regulated choreography allows for a microtubule-directed polarization and release of specialized secretory lysosomes (also called cytolytic granules) into the space between the CTL and target [8]. Among the cytolytic proteins released, perforin and granzymes (Gzms) initiate rapid apoptosis in the target cell [9]. This highly polarized and precise mode of cytotoxicity is essential to prevent aberrant killing of healthy tissue. Given the current interest in therapeutic use of T-cell-mediated toxicity, dissecting the molecular modalities of this process is critical [10].

NFIL3 (nuclear factor IL-3, also called E4BP4) is a mammalian basic leucine zipper transcription factor first identified by its DNA binding activity to the promoter of *IL-3* [11] as well as the ATF consensus site in the adenovirus E4 promoter [12]. NFIL3 is widely expressed in various tissues and has been implicated in a broad range of processes, including the anti-inflammatory response [13], nerve regeneration [14] and the mammalian circadian oscillatory mechanisms [15]. Characterization of *Nfil3^−/−^* mice has revealed diverse roles for NFIL3 in several immune cell types. Among these functions, NFIL3 controls the survival of pro-B lymphocytes [16], drives the development of CD8α^+^ dendritic cells (DCs) [17], modulates the function and stability of regulatory T cells [18], and controls immunoglobulin class switches in B cells [19,20]. Moreover, NFIL3 has a well-established role in natural killer (NK) lineage commitment [21–25]. In CD4^+^ T cells, NFIL3 controls the plasticity of cytokine production. Indeed, regulatory T cells from *Nfil3^−/−^* mice display lower expression of immunoregulatory cytokines IL-10 and IL-13 [26,27]. Additionally, in CD4^+^ T cells, NFIL3 induces the expression of both TIM3, a regulator of T-cell exhaustion, and IL-10 through an IL-27 dependent mechanism [28]. Interestingly, CD8^+^ T-cell development is not impaired in *Nfil3^−/−^* mouse models, but a recent study found that CTLs generated from antigen-activated CD8^+^ T cells clonally expanded in the presence of interleukin 2 (IL-2) display high levels of *Nfil3* mRNA and protein [29]. In humans, the last decade has seen the emergence of a link between NFIL3 and autoimmune diseases such as systemic lupus erythematosus (SLE) [30,31], inflammatory bowel disorders such as Crohn's disease and ulcerative colitis [32], as well as rheumatoid arthritis (RA) and juvenile idiopathic arthritis (JIA) [33–35].

In this study, we performed an RNA-seq analysis of transcriptome changes between naive and effector CD8^+^ T cells and found that *Nfil3* was upregulated during CTL differentiation. Subcellular localization analysis by cell fractionation and immunofluorescence revealed that NFIL3 is constitutively localized in the nucleus of activated CD8^+^ T cells. Using clustered regularly interspaced short palindromic repeats (CRISPR) editing, we found that removing *Nfil3* in CTLs leads to a marked decrease in cytotoxicity. Exploring this defect, we now show that NFIL3 is not required for immune synapse formation and granule release. Strikingly, NFIL3 controls the production of cytolytic proteins as well as effector cytokines. Thus, NFIL3 emerges as a cell intrinsic regulator of CTL-mediated cytotoxicity.

## 2. Results

### 2.1. NFIL3 is expressed following CD8 T-cell differentiation into CTLs

**Figure 1.**
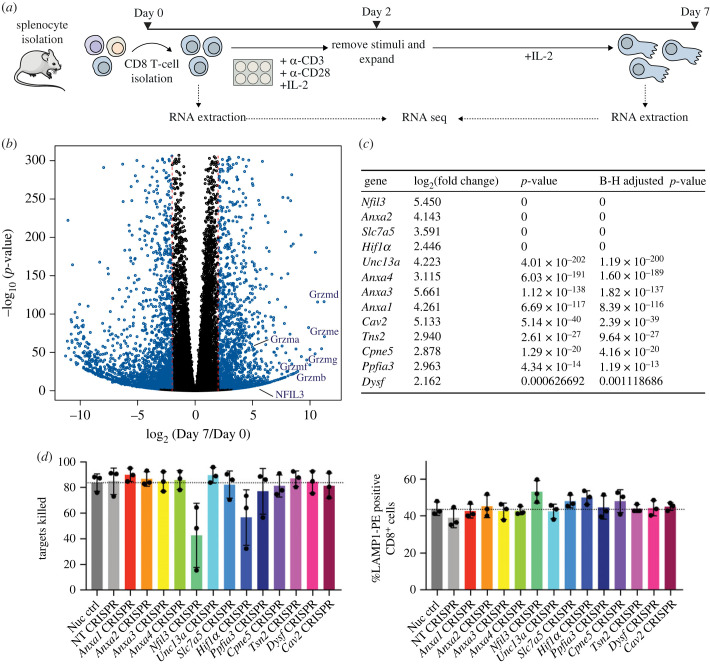
*Nfil3* is expressed following CD8 ^+^ T cell differentiation into CTLs. (*a*) Experimental design for day 0 and day 7 CD8^+^ T-cell isolation from WT C57BL/6N mice, naive CD8^+^ T activation into effector CD8^+^ T cells, and RNA-seq. Ten biological replicates, 4 males and 6 females (16 weeks), were used for this study. (*b*) Volcano plot showing the −log_10_ of the *p*-value (−log_10_(*p*-value), *y*-axis) and the log_2_ of the fold change of expression from day 7 to day 0 (log_2_(Day7/Day0), *x*-axis). Dotted red lines indicate the log_2_(fold change) cut off at −2 and +2. Genes highlighted in blue passed the log_2_(fold change) and were detected as differentially expressed at 1% false discovery rate (FDR) when using Benjamini–Hochberg (BH) multiple testing adjustment. Some genes of interest such as genes encoding members of the granzyme protein family (*Gzme*, *Gzmd*, *Gzmg*, *Gzmb*, *Gzmf*, *Gzma*) and *Nfil3* are indicated in the plot. (*c*) Table showing the differentially expressed genes chosen for the CRISPR screen, their log_2_(fold change) (Day7/Day0), *p*-value and adjusted BH *p*-value. (*d*) CRISPR screen showing T-cell-mediated killing and degranulation (LAMP1^+^ signal) in CD8^+^ T deleted for *Ppfia3*, *Anxa1*, *Anxa2*, *Anxa3*, *Anxa4*, *Tns2*, *Cav2*, *Cpne5*, *Dysf* and *Unc13a*, *Slc7a5*, *Hif1*α and *Nfil3*. Shown as mean and s.d. of three independent experiments performed with two technical replicates each.

T-cell activation begins when the TCR of a naive CD8^+^ T cell recognizes a complex of peptide-major histocompatibility complex class I on an antigen presenting cell (APC) in peripheral lymphoid organs. This initial signalling event induces rapid clonal expansion and effector differentiation. Over several days, the small naive round cells increase in size and go through transcriptional changes, metabolic switches and cytoskeletal rearrangements, all to support their functions as sophisticated cytotoxic cells. To gain better insight into this mechanism, we characterized transcriptomic changes during the differentiation of naive CD8^+^ T cells into cytotoxic T cells (CTLs) by RNA-seq. To this end, we isolated naive CD8^+^ T cells from the spleens of 10 WT mice, 4 males and 6 females (16 weeks). A portion of these cells was used as our day 0 CD8^+^ T cells naive control, while the rest was activated on TCR-specific α-CD3*ε* and α-CD28 antibody-coated plates for 48 h and expanded for 5 days in the presence of IL-2 to generate day 7 cytotoxic T cells (figure 1*a*). The percentage of CD8^+^ T cells was examined on both day 0 and day 7 showing efficient isolation with over 87.5% CD8^+^ cells across all samples (electronic supplementary material, figure S1A). RNA extraction was performed, and RNA quality determined by bioanalyser, with all samples displaying an RNA integrity number between 8.2 and 9.6 (electronic supplementary material, figure S1B). To focus on genes showcasing major changes in transcription, genes were considered differentially expressed when the log_2_(fold change) from day 0 to day 7 was larger than 2 and smaller than −2, and the *p*_adj_ was smaller than 0.01 (figure 1*b*). In total, there were 1802 activated and 2583 repressed genes when comparing day 7 to day 0 (electronic supplementary material, table S1). The top 10 upregulated genes, as ordered by log_2_(fold change) are shown in electronic supplementary material, figure S1C. Among the activated genes we found *Gzmb* and *IL2ra*, that encode Gzm B and the α-subunit of the high affinity IL-2 receptor, respectively (figure 1*b* and electronic supplementary material, figure S1C) [9], that are both important for CTL effector function [29].

To gain a better overall understanding of what biological pathways are enriched in the upregulated gene list, we used the Functional Interpretation of Differential Expression Analysis (FIDEA) together with the Gene Ontology database [36]. We found that cell division, cell cycle, cytoskeleton, and immune system process were among our enriched pathways (electronic supplementary material, figure S1D). From this analysis, 13 genes were chosen to perform a curated CRISPR screen. This screen included 10 genes involved in vesicle fusion (*Ppfia3*, *Anxa1*, *Anxa2*, *Anxa3*, *Anxa4*, *Tns2*, *Cav2*, *Cpne5*, *Dysf* and *Unc13a*) as well as *Slc7a5*, *Hif1*α and *Nfil3* genes as *Slc7a5* deletion was shown to impair CD8^+^ T-cell differentiation, while HIF1α and NFIL3 gene-deletion models displayed defects in perforin production (figure 1*c*) [29,37]. These genes were targeted by CRISPR-mediated deletion in activated CTLs. Cell function was assessed by measuring CTL-mediated killing and degranulation. Strikingly, we found that both *Nfil3* and *Hif1α* deletion led to a reduction in T cell killing. In the case of NFIL3, this was accompanied by a modest increase in degranulation (figure 1*d*). We therefore explored the function of *Nfil3* in CTLs.

### 2.2. NFIL3 is nucleus bound during CTL differentiation

**Figure 2.**
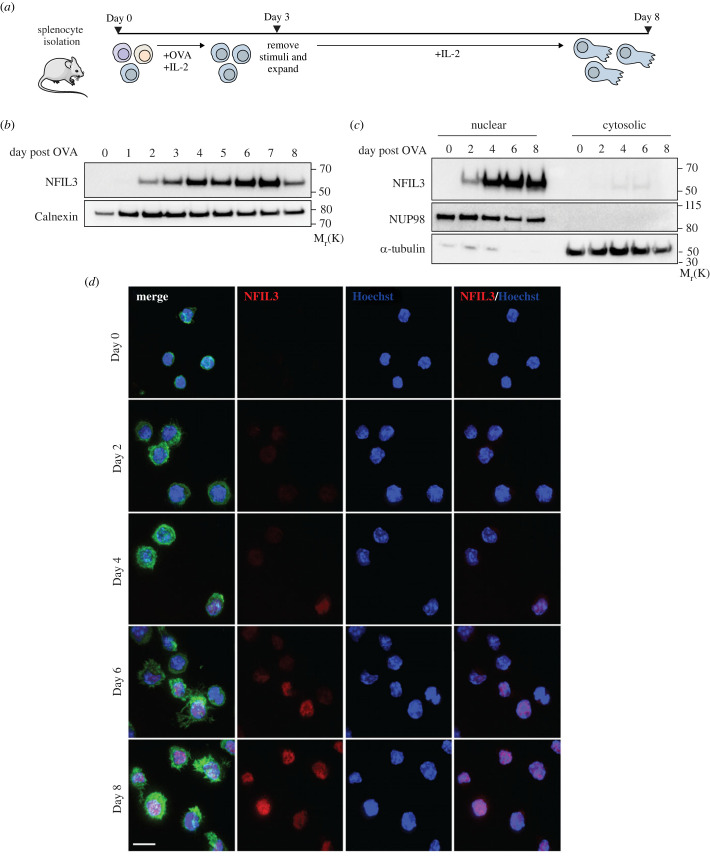
NFIL3 is nucleus bound during CTL development. (*a*) Experimental design for CD8^+^ T cell isolation from OT-I-*Rag1*-deficient mice, naive CD8^+^ T activation into effector CD8^+^ T cells. (*b*) Immunoblotting of OT-I T cells every day from day 0 to day 7 following OVA treatment showing NFIL3 and Calnexin (loading control) protein levels. Molecular markers are indicated. Representative of two biological replicates. (*c*) Immunoblotting of cellular fractionation (nuclear fraction and cytosolic fraction) from OT-I T cells on indicated days following OVA treatment showing NFIL3, NUP98 (nuclear fraction loading control) and α-Tubulin (cytosolic fraction loading control) protein levels. Molecular markers are indicated. Results are representative of three biological replicates. (*d*) Confocal images showing OT-I T cells on indicated days following OVA treatment showing NFIL3 and nucleus localization. Phalloidin (F-actin, green), NFIL3 (red) and Hoechst (nucleus, blue) are shown. Scale bar is 10 µm. Results are representative of two biological replicates.

NFIL3 is a basic leucine zipper transcription repressor capable of modulating gene expression in a wide variety of cellular contexts [38]. Interestingly, we found that *Nfil3* mRNA was significantly upregulated in CTLs between day 0 and day 7 after activation of naive CD8^+^ T cells (figure 1*c*), in accordance with previous proteomic studies [29]. We further confirmed that NFIL3 protein levels were greatly increased in differentiated CD8^+^ T cells compared with naive (electronic supplementary material, figure S1E). We therefore wanted to explore the kinetics of this increase during CTL differentiation as well as the subcellular localization of NFIL3. We used the well-established OT-I-*Rag1*-deficient mouse model in which all peripheral CD8^+^ T cells express a transgenic TCR that recognizes ovalbumin (OVA). Splenocytes were activated with OVA in the presence of IL-2 to generate CTLs (figure 2*a*) and T cells collected at different times. In keeping with our results in WT cells, we found that naive OT-I T cells had undetectable levels of NFIL3 by western blot, and that NFIL3 expression increased following activation (figure 2*b*). We next carried out cellular fractionation and found that surprisingly, NFIL3 was almost exclusively located in the nuclear fraction, independent of differentiation status (figure 2*c*). This was confirmed by immunofluorescence using an antibody against NFIL3 (figure 2*d*). These results show that NFIL3 expression increases steadily during CTL differentiation and this transcription factor is constitutively present in the nucleus of these cells.

### 2.3. NFIL3-depleted CTLs show decreased cytotoxicity

**Figure 3.**
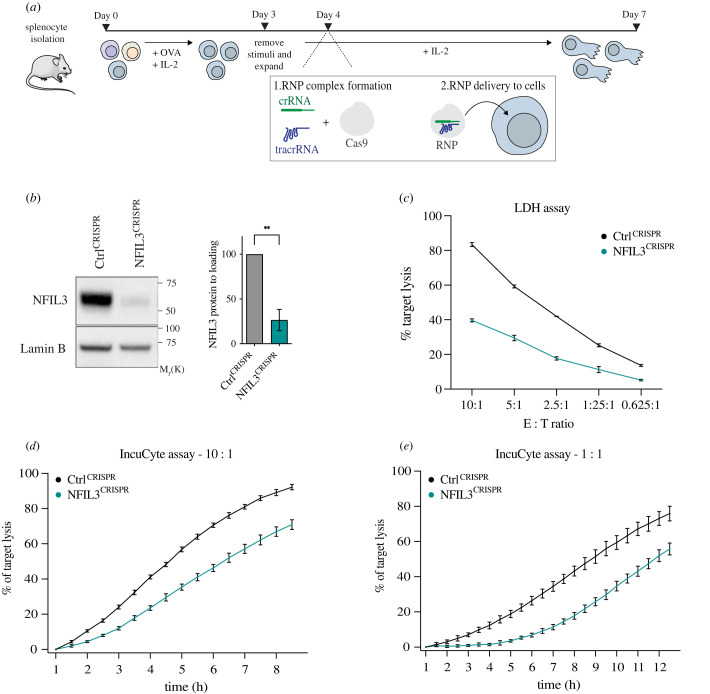
NFIL3-depleted CTLs show decreased cytotoxicity. (*a*) Experimental design for CD8^+^ T cell isolation from OT-I-*Rag1*-deficient mice, naive CD8^+^ T activation into effector CD8^+^ T cells, and ribonucleoprotein CRISPR. (*b–e*) Day 4 CTLs were transfected with RNPs containing a non-targeting crRNA (Ctrl^CRISPR^) or a crRNA specific to *Nfil3* (NFIL3^CRISPR^) and used for functional assay on day 7 (72 h post-CRISPR). (*b*) Immunoblotting and quantification of day 7 Ctrl^CRISPR^ and NFIL3^CRISPR^ CTLs showing NFIL3 and Lamin B (loading control) protein levels. Molecular markers are indicated. Results are representative shown as mean and s.d. of three biological replicates, **p < 0.005 (paired *t*-test). (*c*) Lactate dehydrogenase killing assay with day 7 Ctrl^CRISPR^ and NFIL3^CRISPR^ CTLs and varied number of OVA-presenting EL4 targets for indicated effector : target (E : T) ratios. Per cent of target lysis was measured after 3 h of CTL killing. Shown as mean and s.d. of four technical replicates. Results are representative of three biological replicates. (*d,e*) IncuCyte killing assays with day 7 Ctrl^CRISPR^ and NFIL3^CRISPR^ CTLs and OVA-presenting EL4 targets at E : T ratios of 10 : 1 (*d*) and 1 : 1 (*e*). Per cent of target lysis was measured every 30 min for 8–12 h of CTL killing. Shown as mean and s.d. of four technical replicates. Results are representative of three biological replicates.

Previous work has looked at effector T cells differentiated from CD8^+^ naive cells isolated from *Nfil3^−/−^* knockout mice [29]. However, throughout their lifespan, CD8^+^ T cells go through two major antigen-driven events. The first is APC contact in peripheral lymphoid organs driving differentiation into effectors. The second occurs as CTLs migrate through tissues in search of their cognate target cells. We wanted to dissect the role of NFIL3 in already differentiated CTLs, distinct from its role in differentiation steps. To assess the function of NFIL3, day 4 cells were transfected with CRISPR ribonucleoprotein complexes containing a non-targeting crRNA (Ctrl^CRISPR^) or a crRNA specific for *Nfil3* (NFIL3^CRISPR^) (figure 3*a*). We confirmed that 72 h following CRISPR, NFIL3 protein levels were drastically reduced in NFIL3^CRISPR^ CTLs compared to the control (figure 3*b*). When measuring cytotoxicity, we found that CTLs lacking NFIL3 exhibited reduced killing capacity when challenged with OVA-presenting EL4 targets. In short-term killing assays (3 h), NFIL3^CRISPR^ CTLs only killed about half as many targets as the Ctrl^CRISPR^ for all the effector-to-target ratios tested (figure 3*c*). In long-term assays (8–12 h), NFIL3^CRISPR^ CTLs also displayed reduced killing capacity over time, and this was true at both effector-to-target ratios of 10 : 1 and 1 : 1 (figure 3*d*,*e*). Together, this suggests that NFIL3 has a role in CTL-mediated cytotoxicity, distinct from its role in differentiation.

### 2.4. NFIL3 is not required for immune synapse formation, TCR signalling and granule release

**Figure 4.**
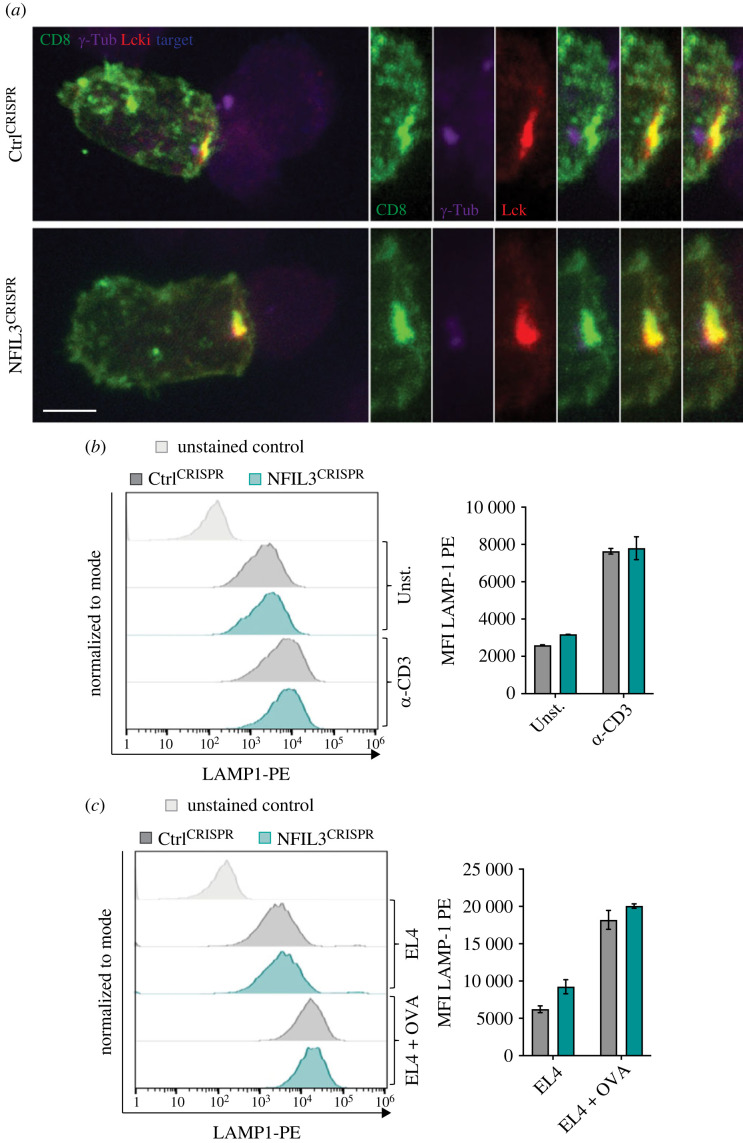
NFIL3 is not required for immune synapse formation, TCR signalling and granule release. (*a–c*) Day 4 CTLs were transfected with RNPs containing a non-targeting crRNA (Ctrl^CRISPR^) or a crRNA specific to *Nfil3* (NFIL3^CRISPR^) and used for functional assay on day 7 (72 h post-CRISPR). (*a*) Confocal images showing day 7 Ctrl^CRISPR^ and NFIL3^CRISPR^ CTLs conjugated with targets. CD8 (green), γ-Tub (violet), Lck (red) and target (blue) are shown. Right panels represent a zoom over the immune synapse. Scale bar is 5 µm. Results are representative of two biological replicates. (*b,c*) Degranulation assay of day 7 Ctrl^CRISPR^ and NFIL3^CRISPR^ on plate-bound α-CD3*ε* (*d*) or with OVA-presenting EL4 targets at E : T ratio 1 : 1 (*e*). LAMP1-PE surface exposure was measured after 3 h, mean fluorescence intensity was quantified. Shown as mean and s.d. of three technical replicates. Results are representative of three biological replicates.

CTL-mediated cytotoxicity is a highly regulated multi-step process. First, CTLs recognize their target cells through their TCR, initiating formation of the immune synapse. There, signalling programmes are engaged, and CTLs go through rapid rearrangements of their cytoskeleton to support the release of cytolytic granules towards the target [4–7]. To gain more insight into the killing defect observed in CTLs lacking NFIL3 we looked at several of these steps. Confocal imaging revealed that NFIL3^CRISPR^ CTLs were able to conjugate with their targets and form an immune synapse (figure 4*a*; electronic supplementary material, figure S2A). Like their control counterparts, NFIL3^CRISPR^ CTLs displayed enriched lymphocyte-specific protein tyrosine kinase (Lck), the kinase that initiates TCR activation, at the contact site, as well as centrosome polarization (figure 4*a*; electronic supplementary material, figure S2A), and TCR signalling was intact with a slight upward trend in extracellular signal-regulated kinase phosphorylation in NFIL3^CRISPR^ CTLs (electronic supplementary material, figure S2B). We then assessed CTL cytotoxic granule release through a lysosome-associated membrane glycoprotein 1 (LAMP1) (also known as CD107a) surface exposure assay. As expected, Ctrl^CRISPR^ CTLs saw an increase in surface LAMP1 when stimulated with plate-bound α-CD3*ε* or placed together with target cells (figure 4*b*,*c*). Interestingly, cells lacking NFIL3 did not present any significant defect in degranulation (figure 4*b*,*c*). Together, this suggests that NFIL3 is dispensable for immune synapse formation and cytotoxic granule release.

### 2.5. NFIL3 is required for perforin and granzyme B expression

**Figure 5.**
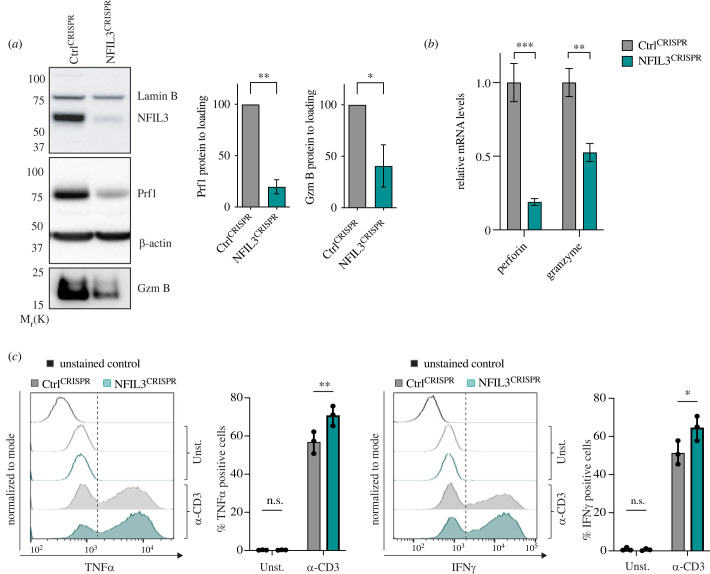
NFIL3 is required for cytolytic protein production. (*a*–*c*) Day 4 CTLs were transfected with RNPs containing a non-targeting crRNA (Ctrl^CRISPR^) or a crRNA specific to *Nfil3* (NFIL3^CRISPR^) and used for functional assay on day 7 (72 h post-CRISPR). (*a*) Immunoblotting and quantification of day 7 Ctrl^CRISPR^ and NFIL3^CRISPR^ CTLs showing NFIL3, perforin, granzyme B and Lamin B and β-actin (loading control) protein levels. Molecular weight markers are indicated. Results are representative of two biological replicates, shown as mean and s.d., **p < 0.005, *p < 0.05 (paired *t*-test). (*b*) qPCR of day 7 Ctrl^CRISPR^ and NFIL3^CRISPR^ CTLs showing *Prf1* and *GzmB* corrected to a GAPDH loading control and normalized to Ctrl^CRISPR^. Shown as mean and s.d. of three technical replicates, ***p < 0.0005, **p < 0.005 (paired *t*-test). Results are representative of three biological replicates. (*c*) Cytokine assay of day 7 Ctrl^CRISPR^ and NFIL3^CRISPR^ stimulated on plate-bound α-CD3*ε* showing % gated cells expressing TNF*α* and IFN*γ*. Shown as mean and s.d. of three technical replicates, **p < 0.005, *p < 0.05, n.s. = non-significant (ANOVA). Results are representative of three biological replicates.

Cytotoxic granules are a form of specialized lysosome filled with cytolytic proteins. Among these are perforin, a pore forming protein that opens channels in the target cell membrane through which Gzms, a family of serine proteases that can activate caspase 3, enter the target to induce apoptosis [9]. We wanted to determine if the cytotoxic defect observed in CTLs lacking NFIL3 was related to modifications of the cytolytic proteins. Strikingly, NFIL3^CRISPR^ CTLs displayed a marked reduction in both perforin and Gzm B protein levels (figure 5*a*), with quantitative PCR revealing decreased mRNA transcription of both Prf1 and GzmB (figure 5*b*). As CD8^+^ effector T cells also produce soluble cytokines that can contribute to cytotoxicity independently of perforin and Gzms [39,40] we assessed the production of TNFα and IFNγ. We found that NFIL3^CRISPR^ CTLs showed increased intracellular TNFα and IFNγ following TCR engagement (figure 5*c*). Interestingly, increased production of IL1-β and TNFα has been reported in an *Nfil3^−/−^* arthritis model [35]. Thus, NFIL3 appears to impact both the production of the cytolytic proteins perforin and Gzms and the production of the cytokines TNFα and IFNγ. These results suggest that NFIL3 plays a role in switching between cytolytic mechanisms in CTLs.

## 3. Discussion

CTLs are important effectors in the clearance of virally infected and cancerous cells, and defects in their function give rise to many pathologies. Moreover, the last few years have seen the emergence of the use of CTLs in clinical approaches [10]. These advances have relied and continue to rely on strong basic science elucidating the molecular and cellular mechanisms involved in both CTL differentiation and activation. Our transcriptomic analysis of changes during the differentiation of CD8^+^ T cells from naive to effector cells has identified a series of upregulated genes (see electronic supplementary material, table S1). Among these we found that *Nfil3* is upregulated in CD8^+^ T effectors. NFIL3 has been shown to be involved in the development of immune cells like NK cells, Treg cells and DCs [38]. Interestingly, while NFIL3 is not required for CD8^+^ T cell differentiation in the presence of IL-2, CTLs differentiated from *Nfil3^−/−^* mice show lower levels of perforin mRNA [29]. Our data demonstrate that NFIL3 is important for cytotoxicity in already differentiated CTLs as CRISPR deletion of NFIL3 in differentiated CTLs resulted in a marked reduction in cytotoxicity although TCR signalling and immune synapse formation were unaffected. The defect appeared to arise from decreased production of perforin and Gzms (both RNA and protein), while the production of cytokines TNFα and IFNγ increased. These results suggest that NFIL3 may play a role in switching between cytolytic mechanisms in CTLs. Intriguingly, increased expression of TNF and IL1-β from *Nfil3^−/−^* mice has been reported in other studies [35]. It is important to note that this role of NFIL3 in negatively regulating TNFα and IFNγ contrasts with its function in CD4^+^ T cells where it drives IL-10 and IL-13 production [26,27]. Determining whether the various context-dependent cytokines that regulate *Nfil3* expression such as IL-2 [29] or IL-4 [19] influence its function will be important moving forward. However, our studies suggest that modulating NFIL3 expression levels could offer a way to dial T-cell-mediated cytotoxicity without affecting events such as immune synapse formation and granule trafficking.

How directly our findings from an *in vitro* system in mice translate into humans remains to be established. NFIL3 has emerged as a regulator of inflammatory pathways involved in several human pathologies. In particular, NFIL3 has been linked to autoimmune disorders such as SLE [30,31], inflammatory bowel diseases (Crohn's disease and ulcerative colitis patients) [32], as well arthritis [33–35]. Strikingly, the protein and mRNA levels of NFIL3 are increased in CD4^+^ T cells isolated from patients with active SLE, and this is modulated by treatment with glucocorticoid [31]. One study found that CD4^+^ T cells isolated from patients suffering from active SLE presented overexpression of NFIL3, increased upon treatment. The use of both silencing and ectopic expression of NFIL3 in CD4^+^ T cells demonstrated a protective role for the transcription factor against the autoimmune response. Interestingly, earlier reports found that CD4^+^ T cells also expressed higher amounts of perforin in patients suffering from active SLE [41]. Even though CD4^+^ T cells have been extensively studied in SLE, the role of CD8^+^ T cells is less well established. Several studies have shown a defect in peripheral blood CTL cytotoxicity in SLE patients, and lupus-prone mice in which CTLs are lacking perforin showed heightened disease progression [42,43]. Whether any of these defects could result from differential NFIL3 expression or post-translational modifications would require further investigation. It will also be important to determine if NFIL3 is required for CD8^+^ T cell differentiation in humans and if the increase in NFIL3 levels we observe from naive to effector T cells in mouse cells is mirrored in humans. Examining the DICE database (database of immune cell expression, expression quantitative trait loci, and epigenomics), NFIL3 expression seems to increase upon CD8^+^ T-cell differentiation, from 18.9 to 42.7 transcripts per million (TPM) [44].

The characterization of monozygotic twin infants that harboured homozygous mutations in NFIL3 established a connection between NFIL3 loss and JIA. Cell lines (LCLs) generated from these patients demonstrated a 50% reduction in NFIL3 protein levels, as compared to their mother [35]. These patients presented defects in both NK and DC lineages, phenocopying the *Nfil3^−/−^* mice phenotype [35]. However, while CD8^+^ T cell numbers are normal in *Nfil3^−/−^* animals, patients had reduced activated CD8^+^ T cells. In both mice and patients, NFIL3 loss drove elevated production of IL-1β in myeloid cells [35]. Interestingly, early studies revealed that CD8^+^ T cells from patients suffering from JIA expressed significantly lower levels of perforin than healthy donors [45]. Similarly, two studies found that NFIL3 expression is linked to RA and put forth the role of NFIL3 in producing inflammatory cytokines [33,34]. The role of CTLs in RA has been debated, with a study finding that expression of Gzm B is increased in the peripheral blood of patients suffering from active RA [46], while another did not [47,48]. Investigating whether CTLs from these patients display any changes in their cytotoxicity will be important.

In summary, we have found that NFIL3 has a dual role on T cell-mediated cytotoxicity through the production of cytolytic proteins and effector cytokines without impeding immune synapse formation. NFIL3 seems to act as a switch between cytolytic mechanisms in CTLs. As NFIL3 is starting to attract attention in the autoimmune field as both a biomarker of disease progression and a therapeutic target [34], the contribution of NFIL3 to some of those disorders could be re-examined.

## 4. Methods

### 4.1. Mice

WT C57BL/6N mice were obtained through the 3i Consortium (Wellcome Trust Sanger Institute, WTSI) and OT-I-*Rag1*-deficient mice (OT-I Rag1tm1Bal on a C57BL/6 N background) were bred and housed in a University of Cambridge facility. This research was regulated under the Animals (Scientific Procedures) Act 1986 Amendment Regulations following ethical review by both the WTSI and the University of Cambridge Animal Welfare and Ethical Review Body (AWERB). Animals ranging from 8 to 16 weeks were used in this study.

### 4.2. Cell culture and reagents

Single cell suspensions of splenocytes from WT C57BL/6N mice, isolated using a nylon 70 µm strainer, were cultured on plates coated with 0.5 µg ml^−1^ α-CD3*ε* (ThermoFisher, clone 500A2) and 1 µg ml^−1^ α-CD28 (ThermoFisher, clone 37.51) for 48 h, resuspended in stimuli free media and further maintained in culture with daily splitting. Single cell suspensions of splenocytes from OT-I-*Rag1*-deficient mice, isolated using a nylon 70 µm strainer, were stimulated with 10 nM of OVA_257–264_ [SIINFEKL] peptide (AnaSpec) for 72 h, resuspended in stimuli free media and further maintained in culture with daily splitting. All mouse T cells were cultured in mouse T-cell media (RPMI 1640, 10% (v/v) FCS, 2 mM glutamine, 1 mM sodium pyruvate, 100 U ml^−1^ penicillin/streptomycin, 50 µM β-mercaptoethanol) supplemented with 20 ng ml^−1^ of recombinant murine IL-2 (PeproTech, 212-12). EL4-NucLight Red cells stably expressing a nuclear restricted red fluorescent label were generated by transducing EL4 cells with IncuCyte NucLight Red Lentivirus (EF1α, puro, Essen Bioscience, 4625) following manufacturer's instructions. After 72 h, cells were sorted by FACS (Becton Dickinson Influx Cell Sorter) and subsequently maintained under continuous selection with 1 µg ml^−1^ of puromycin (ThermoFisher). EL4 expressing Farnesyl-5-TagBFP2 (hereafter called EL4-blue) as previously described in [4] were used for imaging. EL4, EL4-NucLight Red and EL4-blue cells were cultured in DMEM, 10% (v/v) FCS) and 100 U ml^−1^ of penicillin/streptomycin.

### 4.3. RNA-Seq

Ten spleens were collected from 16-week-old WT mice (four males and six females). A mouse CD8a T-cell isolation kit (Miltenyi Biotec, 130-104-075) was used to purify CD8^+^ T cells. 6 × 10^6^ purified cells were washed 2× in ice-cold PBS and frozen at −80°C (day 0 samples). The remainder of the cells were transferred onto plates coated with 0.5 µg ml^−1^ α-CD3*ε* (ThermoFisher, clone 500A2) and 1 µg ml^−1^ α-CD28 (ThermoFisher, clone 37.51) for 48 h, transferred to uncoated plates and split every day. On day 7, 6 × 10^6^ cells were washed 2× in ice-cold PBS and frozen at −80°C (day 7 samples). On both day 0 and day 7, CD8 expression levels were analysed by flow cytometry.

Total RNA was extracted from pelleted cells using QIAshredder columns and RNeasy mini kit (Qiagen) following manufacturer instructions. Concentration and quality of extracted RNA were assessed using a Qubit fluorometer (ThermoFisher) and a bioanalyser (Agilent), respectively. ERCC RNA Spike-In controls (Ambion, Life Technologies) were added to the samples following the manufacturer instructions. 1 µg of RNA per sample was submitted to the Illumina bespoke team at WTSI. Stranded 75 base pair (bp) paired-end barcoded libraries were generated using oligodT pulldown and sequencing was performed on an Illumina Hiseq platform. Data analysis followed a published workflow [49]. Briefly, reads were aligned to the mouse reference genome (GRCm38) using STAR (v2.5.0a), a splice aware mapper, guided by the ENSEMBL mouse annotation (v84) [50]. To assess gene expression, reads were counted using HTSeq (v0.5.4p3) [51]. Raw counts were transformed to TPM. Unsupervised hierarchical clustering of samples and principal component analysis were performed as quality control examinations. The DESeq2 (v1.10.1) Bioconductor package was used to identify differentially expressed genes between day 0 and day 7 [49]. A multi-factor design where sex and spleen collection date were included alongside day after activation group was used. *p*-values were corrected for multiple testing using the Benjamini–Hochberg method to give adjusted *p*-values (padj). Genes are considered differentially expressed with a log_2_(fold change) (Day7/Day0) < −2 or greater than 2 and a padj < 0.01. Sequencing data are available through the European Nucleotide Archive (ENA) with study accession number ERP107420.

### 4.4. Immunoblotting

Cells were washed with ice-cold PBS prior cell lysis. For total cell extracts, cells were lysed with TNT buffer (50 mM Tris–HCl (pH 7.4), 150 mM NaCl, 1% (v/v) NP-40, 1% (v/v) Triton X-100, 1 mM EDTA) supplemented with Halt protease inhibitor cocktail (ThermoFisher) on ice for 30 min and samples were cleared by centrifugation at 12 000*g*. For cell fractionations, 10 × 10^6^ cells were lysed in 87.5 µl of Buffer A (10 mM Hepes (pH 7.9), 10 mM KCl, 0.1 mM EDTA, 0.1 mM EGTA, 1 mM DTT, 1 mM Na_3_VO_4_) supplemented with Halt protease inhibitor cocktail on ice for 5 min. 12.5 µl of initial volume of Buffer A containing 10% NP-40 was added for another 5 min and followed by centrifugation at 2500*g* for 3 min. Pellets containing nucleus were further spun on a sucrose gradient (Nuclei Pure Sucrose Cushion, Sigma) at 20 000*g* for 45 min to obtain the nuclear fraction while supernatants were cleared as above to obtain cytosolic fraction. The resulting nuclear pellets were lysed in 30 µl of Buffer C (20 mM Hepes (pH 7.9), 400 mM NaCl, 1 mM EDTA, 1 mM EGTA, 1 mM DTT, 1 mM Na_3_VO_4_) supplemented with Halt protease inhibitor cocktail on ice for 30 min and cleared by a 10 000*g* centrifugation. Protein concentration was determined by BCA (ThermoFisher, 23 235). In total, 5–10 µg of proteins were loaded with 4× NuPage sample buffer (ThermoFisher, NP0007), resolved by NuPAGE gels (ThermoFisher) and transferred to 0.2 µm nitrocellulose membranes (Bio-Rad). Membranes were blocked and incubated with antibodies using 5% milk, washed in TBS supplemented with 0.1% Tween and revealed using Immobilon western chemiluminescent HRP substrate (Millipore) on a ChemiDoc MP Imaging System (Bio-Rad). To assess signalling, OT-I cells were stimulated with 0.5 µg ml^−1^ of plate-bound α-CD3*ε* (ThermoFisher, clone 500A2) for indicated times at 37°C prior to lysis.

The following antibodies were used for immunoblotting: anti-NFIL3/E4BP4 (Cell Signalling, D5K8O) [1 : 5000], anti-pErk1/2 (p44/42 mitogen-activated protein kinase (MAPK)) (Thr202/Tyr204) (Cell Signalling, D13.14.4E) [1 : 1000], anti-Erk1/2 (p44/42 MAPK) (Cell Signalling, 9102) [1 : 5000], anti-GAPDH (Santa Cruz, 6C5, sc-32233) [1 : 20 000], anti-Granzyme B (Abcam, ab4059) [1 : 1000], anti-Perforin (Enzo, CB5.4) [1 : 500], anti-Calnexin (Sigma) [1 : 2000], anti-Lamin B1 (Abcam, ab16048) [1 : 5000], anti-NUP98 (Cell Signalling, C39A3) [1 : 2000]. Species and isotype relevant secondary antibodies coupled with HRP from Southern Biotech were used at [1 : 5000].

### 4.5. CRISPR

TrueCut Cas9 Protein v2 (A36499, ThermoFisher), tracrRNA (U-002005, Dharmacon) and either non-targeting crRNA (U-007501-01) or 3 guide crRNA against *Nfil3* (CM-063246-01, CM-063246-02, CM-063246-03, Dharmacon) were mixed at a 1 : 1 : 1 (200 µM) ratio in P3 Primary Cell Nucleofector Solution (V4XP-3024, Lonza). Day 4 OT-I CTLs were washed in PBS, resuspended in the solution mix, nucleofected using the unstimulated mouse T-cell program on the Amaxa 4D-Nucleofector and transferred into pre-warmed recovery media (RPMI, 5% (v/v) FCS, 2 mM glutamine, 1.7 mM sodium pyruvate, 32 µM 1-thioglycerol, 20 µM bathocuproinedisulfonic acid disodium salt) for 4 h at 37°C before splitting in mouse T cell media. Cells were resuspended in fresh media 24 h following nucleofection.

### 4.6. Immunofluorescence

OT-I T cells were collected at days shown following activation and pipetted onto 5-well slides (Hendley, P299) and incubated for 10 min at 37°C. Cells were fixed in 4% PFA for 15 min, incubated with 0.1% Triton X-100, blocked in PBS 1% BSA for 30 min at room temperature, and incubated with primary and secondary antibodies in PBS 1% BSA at room temperature for 2 and 1 h, respectively, then 5 min in Hoechst [1 : 50 000 in PBS] to stain the nucleus.

CTLs (7 days after activation and 72 h post-CRISPR) and EL4-blue (previously pulsed with 1 µM of OVA_257–264_ for 1 h at 37°C and washed 3×) were resuspended at a 1 : 1 effector-to-target (E : T) ratio (10^6^ cells ml^−1^) in serum free mouse media. CTLs and targets were allowed to form conjugates at 37°C for 5 min before being pipetted on 5-well slides using cut-off pipette tips and further incubated 15 min at 37°C. Cells were fixed using ice-cold methanol on a cold tray for 2 min, blocked in PBS 1% BSA for 30 min at room temperature, and incubated with primary and secondary antibodies in PBS 1% BSA at room temperature for 2 and 1 h, respectively.

Cells were washed thoroughly in PBS between staining steps. Slides were mounted in ProLong Diamond Antifade mounting media (ThermoFisher, P36961) with a no. 1 coverslip and imaged at room temperature using an Andor System with an Olympus IX81S1F-3-5 body, Yokogawa CSU-X1 spinning disc, and an iXon Ultra 888 camera. Olympus Plan Apochromat 100 X 1.4 NA objective oil was used, and *z*-stacks were collected with a *z*-step distance of 0.2 µm. Images were acquired using the Fusion software and rendered using Imaris 9 software (Bitplane).

The following antibodies were used for immunofluorescence: anti-NFIL3 (BioLegend, clone 12H8C35) [1 : 100], anti-γTubulin (Sigma, T5192) [1 : 400], anti-Lck (Merck Millipore, clone 3A5, 05-435) [1 : 100] and anti-CD8a-AF488 (clone 53-6.7, 14-0081-82) [1 : 400]. Secondaries Alexa Fluor 546-conjugated anti-mouse IgG and Alexa Fluor 647-conjugated anti-rabbit IgG from ThermoFisher were used at [1 : 400].

### 4.7. Flow cytometry, degranulation assay and cytokine assay

Day 0 and day 7 WT CTLs were suspended in ice-cold FACS buffer (PBS, 2% (v/v) FCS) and labelled with anti-mouse CD8a APC (BioLegend, 53-6.7) for 20 min at 4°C and washed in PBS before analysis.

CTLs (7 days after activation and 72 h post-CRISPR) were incubated at a 1 : 1 E : T ratio with EL4 (previously pulsed with 1 µM of OVA_257–264_ for 1 h at 37°C and washed 3× in mouse T-cell media) or on 0.5 µg ml^−1^ of plate-bound α-CD3*ε* (ThermoFisher, clone eBio500A2) in the presence of rat anti-mouse CD107a-PE (eBioscience, clone 1D-4B) for 3 h at 37°C. Cells were suspended in ice-cold FACS buffer (PBS, 2% (v/v) FCS) and labelled with Brilliant Violet 711 anti-mouse CD8a (BioLegend, clone 53-6.7) and a Zombie viability dye (BioLegend) for 20 min at 4°C, and washed in PBS.

CTLs were counted and resuspended in mouse T-cell medium at a concentration of 5 × 10^5^ ml^−1^. One millilitre of cells was then added to wells of a 12-well polystyrene plate that had been coated with PBS alone (unstim.) or 5 µg ml^−1^ anti-mouse CD3*ε* (ThermoFisher, 14-0033-82, clone 500A2) in PBS. The plate was incubated at 37°C, 8% CO_2_ for 1 h. At the end of the incubation, the plate was placed on ice and the wells were resuspended and transferred to tubes. Cells, including an unstained control sample, were centrifuged (300*g*, 5 min), supernatant aspirated, and pellet resuspended in 150 µl of ice-cold PBS with 1 : 400 LIVE/DEAD Fixable Yellow Dead Cell Stain (ThermoFisher, L34959). Staining proceeded for 20 min on ice in the dark. During staining, each sample was split into three wells of a 96-well plate on ice. Using the Foxp3/Transcription Factor Staining Buffer Set (ThermoFisher, 00-5523-00) according to the manufacturer's protocol, cells were fixed and stained for cytoplasmic cytokines with anti-mouse TNF*α* conjugated to AlexaFluor-488 (BioLegend, 506313, clone MP6-XT22) and anti-mouse IFNγ conjugated to phycoerythrin (BioLegend, 505808, clone XMG1.2), both at 1 : 200 dilution. Fixation was performed for 20 min at room temperature, and staining was performed for 60 min at room temperature. Cells were finally resuspended in flow cytometry buffer (1% FCS in PBS) and analysed with an Attune NxT flow cytometer, acquiring with VL-3, BL-1 and YL-1 filter sets. Flow cytometry data were acquired on LSRFortessa (BD Biosciences) and Attune NxT (ThermoFisher) and analysed using FlowJo v.10 (FlowJo, LLC).

### 4.8. Killing assays

For lactate dehydrogenase assay, OT-I CTLs (7 days after activation and 72 h post-CRISPR) were washed and resuspending in killing assay media (phenol red-free RPMI and 2% (v/v) FCS) and mixed at indicated E : T ratios with EL4 (previously pulsed with 1 µM of OVA_257–264_ for 1 h at 37°C and washed 3× in killing assay media) in a round-bottomed 96-well plate for 3 h at 37°C. The percentage of target lysis was measured using the CytoTox 96 Non-Radioactive Cytotoxicity Assay (Promega).

For the IncuCyte assay, OT-I CTLs (7 days after activation and 72 h post-CRISPR) were mixed at 10 : 1 and 1 : 1 E : T ratios with EL4-NucLight Red (previously pulsed with 1 µM of OVA_257–264_ for 1 h at 37°C and washed 3× in mouse T cell media). CTLs were carefully pipetted on top of pelleted targets in an ultra-low attachment round-bottomed 96-well plate (7007, Corning). Plates were scanned using the 4× objective lens of an IncuCyte S3 Live Cell Analysis (Sartorius) in both the red and brightfield channels every 30 min for 8–12 h. Target lysis was assessed by quantifying the loss of red fluorescence using the IncuCyte S3 software and the spheroid assay module (Sartorius).

### 4.9. RNA extraction and real-time quantitative PCR

Total RNA was extracted from OT-I CTLs using QIAshredder columns and RNeasy mini kit (Qiagen) following manufacturer instructions. One microgram of RNA was reverse transcribed using a High-Capacity RNA-to-cDNA kit (ThermoFisher) and gene expression was measured using a TaqMan Gene Expression Assay (ThermoFisher) and a CFX96 Touch Real-Time PCR Detection System (Bio-Rad). Data were analysed using the 2^−ΔΔCT^ method and corrected by the house keeping gene. The following probes were used: mouse GAPDH probe Mm99999915_g1, mouse Perforin probe Mm00812512_m1, mouse Granzyme B probe Mm00442837_m1 (ThermoFisher).

### 4.10. Quantification and statistical analysis

The numbers of technical and independent biological repeats are specified in figure captions. Results are shown as mean and s.d. Statistical analysis was performed using GraphPad Prism 9 and results are described in figure captions.

## Data Availability

Data are released from the study Sequencescape Sequencing StudyID 4082 which shows ENA Study Accession Number as: ERP107420. Supplementary material is available online [52].
